# The complete mitochondrial genome of false trevally *Lactarius Lactarius* (Bloch and Schneider, 1801)

**DOI:** 10.1080/23802359.2019.1698335

**Published:** 2019-12-11

**Authors:** Min Yang, Pengfei Li, Qiwei Qin, Kecheng Zhu

**Affiliations:** a Joint Laboratory of Guangdong Province and Hong Kong Region on Marine Bioresource Conservation and Exploitation, College of Marine Sciences, South China Agricultural University, Guangzhou, China;; b Guangxi Key Lab for Marine Biotechnology, Guangxi Institute of Oceanography, Guangxi Academy of Sciences, Nanning, P. R. China;; c Guangxi Key Laboratory of Marine Environmental Science, Guangxi Academy of Sciences, Nanning, P. R. China;; d Key Laboratory of South China Sea Fishery Resources Exploitation and Utilization, Ministry of Agriculture, South China Sea Fisheries Research Institute, Chinese Academy of Fishery Sciences, Guangzhou, China

**Keywords:** *Lactarius lactarius*, mitochondrial, genome

## Abstract

In the present study, the complete mitochondrial DNA sequence of the milkfish *Lactarius lactarius* is determined. The full-length of the mitochondrial genome consists of a 16,552 bp fragment, with the base composition of A (28.24%), C (29.82%), G (15.96%), and T (25.98%) with a high A + T content (54.22%). The base compositions present clearly the A-T skew, which is most obvious in the control region and protein-coding genes. It includes 2 ribosomal RNA (rRNA) genes, 13 protein-coding genes (PCGs), 22 transfer RNA (tRNA) genes, and a major non-coding control region (D-loop region). Furthermore, the composition and order of these genes are identical to most of other vertebrates. All the protein initiation codons are ATG, except that *COX1* and *ATP6* begin with GTG. All the protein termination codons are TAA, except *ND3* and *ND6* end with TAG, and *COXII*, *ND4*, *Cytb* finish only with incomplete T. Furthermore, the phylogenetic analysis base on 13 concatenated PCGs amino acid datasets among the 14 species, suggesting that high value support for the following sister clade with *Toxotes chatareus*. Our findings provide useful information for phylogenetic and evolutionary research of Perciformes species.

The false trevally *Lactarius lactarius*, is unique fish from *Lactarius*, *Lactariidae*, Perciformes. It distributes generally in the Indian Ocean-western and coastal area of China (Leis 1999). This species of habitat type is marine neritic and the extent and quality of habitat is ceaselessly decline in area. The present population trend of *L. lactarius* is unknown (Kaymaram et al. 2015) and is only known that partial of fish distributes in the Persian Gulf from a trawl sample off Iran (Valinassab et al. 2006). In the present study, we characterize the complete mitochondrial genome DNA (mtDNA) sequence of *L. lactarius* for phylogenetic analysis.

The samples of *L. lactarius* are collected from Hailing island, Yangjiang city, Guangdong province, China (111°52′00″E, 21°40′00″N). The specimens now are deposited in the South China Sea Fisheries Research Institute (Guangzhou, China). The genomic DNA extracts from standard phenol/chloroform method (Sambrook and Russel 2000). The method for preparing and sequencing the DNA library is referred to Zhu, Liang, et al. (2017). For mtDNA sequence assemble, annotation and analysis are referred to Zhu, Wu, et al. (2017). All the specimens (Accession number: Ssfri-F00156) and the genomic DNA are deposited in the Tropical and Subtropical Marine Life Museum of South China Sea Fisheries Research Institute, Guangzhou, Guangdong, China.

The complete mtDNA of *L. lactarius* is performed to be 16,552 bp in length (Accession number: MN604078), with the following nucleotide composition: A (28.24%), T (25.98%), C (29.82%), G (15.96%), and with a high A + T content (54.22%). Moreover, A (32.31%), T (34.08%), C (19.29%), and G (14.32%) are in D-loop region. There are ten overlapping regions, ranging from 1 to 10 bp. And 11 intergenic regions exist in this genome, ranging from 1 to 40 bp. Furthermore, the gene content is equally to other Perciformes mtDNA (Yagishita et al. 2009; Zhang et al. 2016; Zhu, Wu, et al. 2017), comprising 13 protein coding genes (PCGs), 22 tRNAs, 2 rRNAs, and a control region.

Only one PCGs (*ND6*), and eight tRNA genes (Gln, Ala, Asn, Cys, Tyr, Ser, Glu, and Pro) are encoded by L strand, while two rRNA genes (12S rRNA and 16S rRNA), twelve PCGs (*ND1*, *ND2*, *COX1*, *COXII*, *ATP8*, *ATP6*, *COXIII*, *ND3*, *ND4L*, *ND5*, and *Cytb*), fourteen tRNA genes (Phe, Val, Leu, Ile, Met, Trp, Asp, Lys, Gly, Arg, His, Ser, Leu, and Thr), and a non-coding region (D-loop region) are encoded by H strand. Eleven of thirteen PCGs start with the representative initiation codon ATG, only *COX1* and *ATP6* with GTG. Eight PCGs initial TAA and *ND3*, *ND6* use TAG as stop codon, whereas *COXII*, *ND4*, and *Cytb* use incomplete stop codon T-. It is analogously with that in *Toxotes chatareus*, *Elagatis bipinnulata*, and *Lutjanus carponotatus* mtDNA (Yagishita et al. 2009; Ma et al. 2017; Kim et al. 2019).

There are 22 tRNAs genes in the mtDNA of *L. lactarius*, ranging from 67 bp (tRNA^Cys^) to 75 bp (tRNA^Lys^). Non-coding region is A + T enrichment region, which is related to transcription and replication (Clayton 1992). The non-coding region of *L. lactarius* is located between tRNA^Pro^ and tRNA^Phe^ genes, is 845 bp long with high A + T content (66.39%). Furthermore, all the tRNAs can be folded into the representative cloverleaf secondary structures, except tRNA^Ser(GCT)^ which lacks dihydrouridine (DHU) arm.

To investigate phylogenetic relationships between Centrarchiformes, Perciformes, Lutjaniformes, Carangiformes, and Istiophoriformes, a phylogenetic tree is constructed by MEGA 7.0 based on 13 tandem PCGs amino acid sequences with Maximum likelihood (ML) method and MtMam + I + G + F model (Kumar et al. 2016). Because of both two species are belong to Perciformes, *T. chatareus* is grouped with *L. lactarius* as the sister species (Figure 1). The *L. lactarius* mtDNA will provide additional genetic information for genetic analyses in the future study.

**Figure 1.**
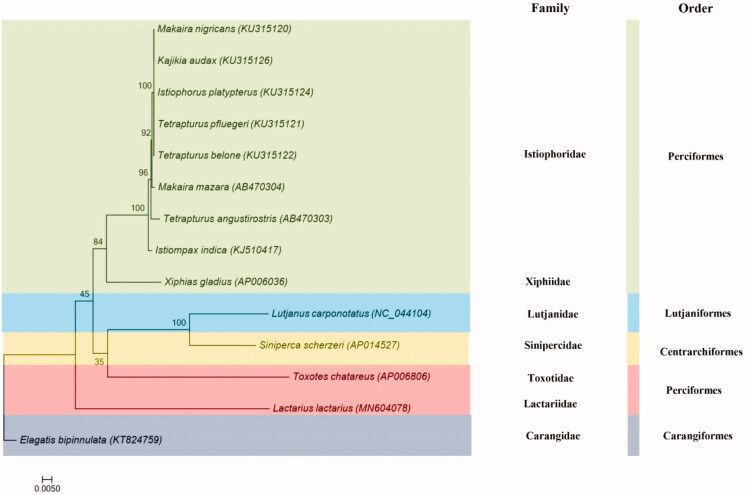
Phylogenetic trees of *L. lactarius* relationships from the amino acid datasets. Sequence alignment of 13 PCGs was analyzed using the MEGA 7.0 with ML method. The accession numbers of the sequences used in the phylogenetic analysis are showed in Figure.

## References

[CIT0001] Clayton DA. 1992. Transcription and replication of animal mitochondrial DNAs. Int Rev Cytol. 141:217–232. 1452432 10.1016/s0074-7696(08)62067-7

[CIT0002] Kaymaram F, Abdulqader E, Al-Husaini M, Hartmann S, Alam S, Almukhtar M, Alghawzi Q. 2015. *Lactarius lactarius*. The IUCN Red List of Threatened Species 2015: e.T46086218A57139758. Arizona: Arizona State University.

[CIT0003] Kim G, Lee JH, Alam MJ, Lee SR, Andriyono S. 2019. Complete mitochondrial genome of Spanish flag snapper, *Lutjanus carponotatus* (Perciformes: Lutjanidae). Mitochondrial DNA B Resour. 4(1):568–569.

[CIT0004] Kumar S, Stecher G, Tamura K. 2016. MEGA7: molecular evolutionary genetics analysis Version 7.0 for bigger datasets. Mol Biol Evol. 33(7):1870–1874. 27004904 10.1093/molbev/msw054 PMC8210823

[CIT0005] Leis J. 1999. Lactariidae. In: Carpenter K. and Niem V. editors. The living marine resources of the western-central pacific. Rome: FAO; p. 2649.

[CIT0006] Ma C, Ma H, Zhang H, Feng C, Wei H, Wang W, Chen W, Zhang F, Ma L. 2017. The complete mitochondrial genome sequence and gene organization of the rainbow runner (*Elagatis bipinnulata*) (Perciformes: Carangidae). Mitochondrial DNA A DNA. 28(1):5–6. 10.3109/19401736.2015.1106516 26709669

[CIT0007] Sambrook JD, Russell W. 2000. Molecular cloning: a laboratory manual. 2nd ed. Cold Spring Harbor, New York: Cold Spring Harbor Laboratory Press.

[CIT0008] Valinassab T, Daryanabard R, Dehghani R, Pierce GJ. 2006. Abundance of demersal fish resources in the Persian Gulf and Oman Sea. J Mar Biol Ass. 86(6):1455–1462.

[CIT0009] Yagishita N, Miya M, Yamanoue Y, Shirai SM, Nakayama K, Suzuki N, Satoh TP, Mabuchi K, Nishida M, Nakabo T. 2009. Mitogenomic evaluation of the unique facial nerve pattern as a phylogenetic marker within the percifom fishes (Teleostei: Percomorpha). Mol Phylogenet Evol. 53(1):258–266. 19540351 10.1016/j.ympev.2009.06.009

[CIT0010] Zhang DC, Wang L, Guo H, Ma Z, Zhang N, Lin J, Jiang SG. 2016. Complete mitochondrial genome of Florida pompano *Trachinotus carolinus* (Teleostei, Carangidae). Mitochondrial DNA A. 27(1):597–598. 10.3109/19401736.2014.908363 24730608

[CIT0011] Zhu KC, Liang YY, Wu N, Guo HY, Zhang N, Jiang SG, Zhang DC. 2017. Sequencing and characterization of the complete mitochondrial genome of Japanese Swellshark (*Cephalloscyllium umbratile*). Sci Rep. 7(1):15299. 29127415 10.1038/s41598-017-15702-0 PMC5681689

[CIT0012] Zhu KC, Wu N, Sun XX, Guo HY, Zhang N, Jiang SG, Zhang DC. 2017. Characterization of complete mitochondrial genome of fives tripe wrasse (*Thalassoma quinquevittatum*, Lay and Bennett, 1839) and phylogenetic analysis. Gene. 598:71–78. 27816474 10.1016/j.gene.2016.10.042

